# Distinct Functional Constraints Partition Sequence Conservation in a *cis*-Regulatory Element

**DOI:** 10.1371/journal.pgen.1002095

**Published:** 2011-06-02

**Authors:** Antoine Barrière, Kacy L. Gordon, Ilya Ruvinsky

**Affiliations:** 1 Department of Ecology and Evolution and Institute for Genomics and Systems Biology, Chicago, Illinois, United States of America; 2 Department of Organismal Biology and Anatomy, The University of Chicago, Chicago, Illinois, United States of America; University of California Berkeley, United States of America

## Abstract

Different functional constraints contribute to different evolutionary rates across genomes. To understand why some sequences evolve faster than others in a single *cis*-regulatory locus, we investigated function and evolutionary dynamics of the promoter of the *Caenorhabditis elegans unc-47* gene. We found that this promoter consists of two distinct domains. The proximal promoter is conserved and is largely sufficient to direct appropriate spatial expression. The distal promoter displays little if any conservation between several closely related nematodes. Despite this divergence, sequences from all species confer robustness of expression, arguing that this function does not require substantial sequence conservation. We showed that even unrelated sequences have the ability to promote robust expression. A prominent feature shared by all of these robustness-promoting sequences is an AT-enriched nucleotide composition consistent with nucleosome depletion. Because general sequence composition can be maintained despite sequence turnover, our results explain how different functional constraints can lead to vastly disparate rates of sequence divergence within a promoter.

## Author Summary

Comparison between genome sequences of different species is a powerful tool in modern biology because important features are maintained by natural selection and are therefore conserved. However, some important sequences within genomes evolve considerably faster than others. One possible explanation is that they encode little or no function. Alternatively, they may evolve under different constraints that permit sequence turnover while maintaining function. Here we report that the promoter of the *unc-47* gene of *C. elegans* contains two discrete elements. One has a highly conserved sequence that determines the spatial expression pattern. Another shows no sequence conservation, but it makes expression of the gene robust, that is, consistent between individuals and resilient to environmental challenges. Remarkably, multiple unrelated sequences are capable of promoting robust expression. Nucleotide composition of these sequences suggests that open chromatin may play a role in conferring robustness of gene expression. Because general sequence composition and therefore expression robustness can be maintained despite sequence turnover, our results offer an explanation of how rapidly diverging promoter elements can nevertheless remain functionally conserved.

## Introduction

The advent of genome sequencing introduced the practice of searching for regulatory elements in evolutionarily conserved regions [1]–[5]. However, functional elements are by no means strictly confined to regions of high primary sequence conservation [6]–[9]. In fact, *cis*-regulatory elements can retain functionality over great evolutionary distances despite sharing little or no identifiable sequence similarity, and can correctly drive reporter gene expression when placed in a distantly related species [10]–[12].

Two questions arise from these observations. First, how do different functional constraints account for different degrees of sequence conservation? Whereas the relationship between function and sequence conservation in not well understood in general, this problem is particularly acute for *cis*-elements [13]. A major obstacle is that we do not have a *cis*-regulatory code akin to that for protein-coding sequences. For example, even within conserved *cis*-regulatory elements there are interspersed nonconserved sequences that seem to be important for their function [12], [14]–[18]. In other cases, *cis*-regulatory architecture can be cryptically conserved despite sequence divergence [19]–[21]. In yet other promoters, not even the architecture appears to be conserved [22], [23].

Second, since gene expression is increasingly considered to be a quantitative trait for which populations vary [24], [25], functional comparisons of regulatory elements ought to be made with quantitative measurements across populations of individuals [26] or cells [27]. Only then can expression patterns be compared in terms of how much they differ, and how intrinsically variable they are.

Variation of gene expression can take many forms, for instance the number of cells expressing a gene or the amount of transcript made in individual cells. Despite variation, gene expression, like many other biological processes, exhibits substantial robustness, that is, resilience to perturbations by genetic and environmental challenges [28]–[31]. Robustness of expression, much like pattern of expression, is encoded in regulatory elements [32], [33]. One way of encoding robustness in *cis* is with redundant or “shadow” enhancers [34]. The loss of one “shadow” enhancer does not substantially perturb gene expression, unless the organism is challenged by genetic or environmental stresses [35], [36]. Another documented mechanism that confers robustness in *cis* is the presence of miRNA target sites in 3′ UTR [37], [38].

Our goal is to understand the relationship between function and sequence evolution in a single *cis*-element. We studied a promoter of the *Caenorhabditis elegans unc-47* gene, which drives a simple, easily quantifiable expression pattern. This promoter contains regions of high and low sequence conservation when compared to orthologs from four closely related [39] *Caenorhabditis* nematodes. We quantified functional similarities and differences of these promoters to infer the constraints that gave rise to the observed patterns of sequence evolution.

## Results

### Conserved *cis*-elements recapitulate qualitative aspects of the expression pattern

**Figure 1:**
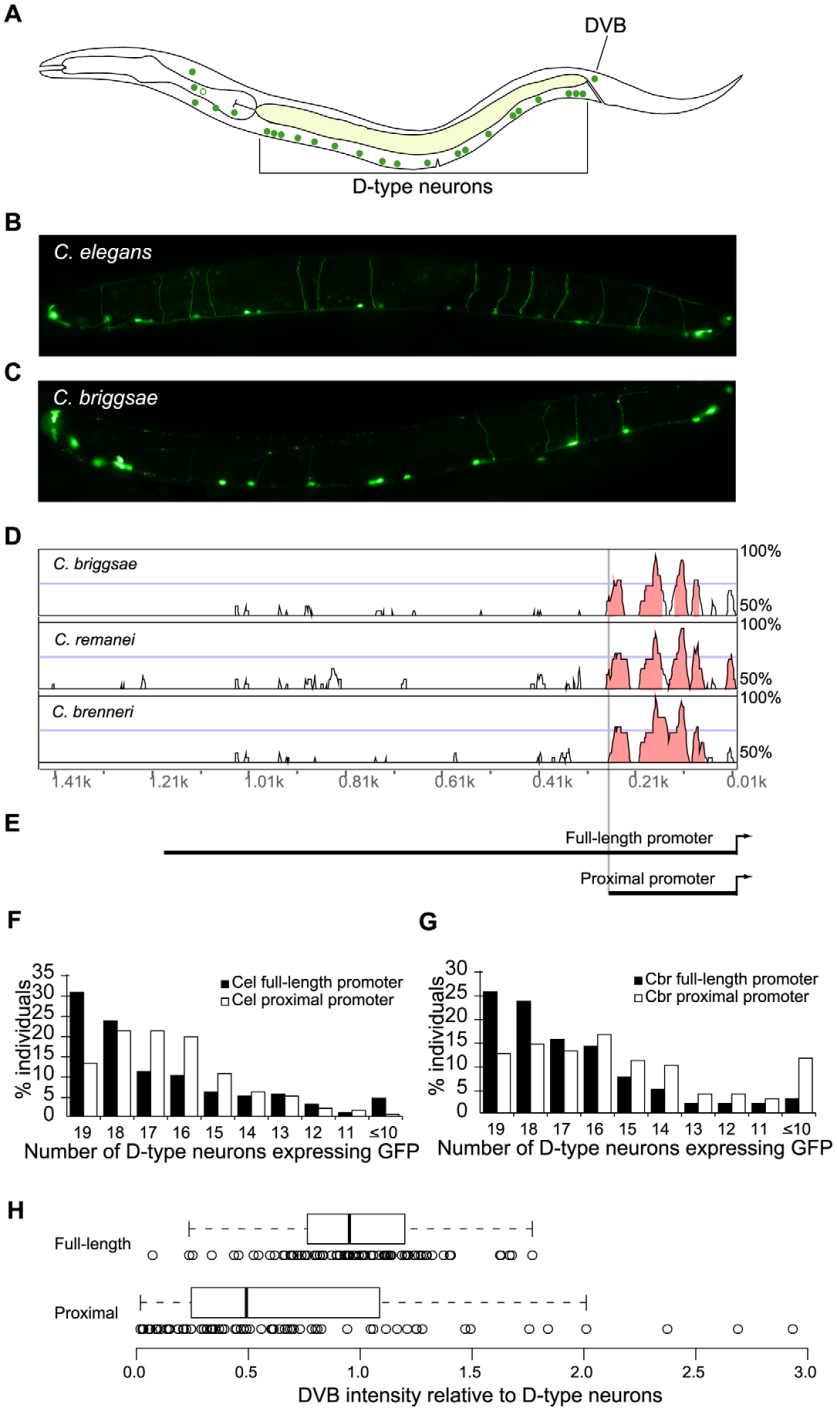
Conserved portions of *unc-47* promoters direct spatially correct, but not robust, expression. (A) A schematic depiction of GABAergic neurons in *C. elegans*. (B) Expression pattern of *C. elegans* promoter *unc-47*::GFP in *C. elegans*. (C) Expression pattern of *C. briggsae* promoter *unc-47*::GFP in *C. briggsae*. Photographs of worms are composites of multiple exposures of the same individual that capture the full complement of D-type neurons, in all focal planes, expressing GFP. (D) Vista plot of primary sequence conservation in the promoter region of *unc-47* from *C. briggsae*, *C. remanei* and *C. brenneri* aligned to *C. elegans*. Window size = 20 bp, threshold = 70%. (E) Schematic depiction of full-length and proximal promoters. Consistency of GFP expression in D-type neurons for full-length and proximal promoters from *C. elegans* (F) and *C. briggsae* (G). For both *C. elegans* and *C. briggsae*, the average number of cells expressing GFP is lower for the proximal promoter compared to full-length (Wilcoxon test, p = 3.2×10^−3^ and p = 3.4×10^−8^, respectively). (H) Distribution of ratios of GFP expression intensity in DVB relative to D-type neurons for the full-length and proximal promoters at 20°C. Each strain is represented by 100 animals. The two distributions are significantly different (Wilcoxon test, p = 1.8×10^−5^; Kolmogorov-Smironov test, p = 1.1×10^−8^).

We first tested the hypothesis that an evolutionarily conserved expression pattern results from evolutionarily conserved regulatory sequences alone. In *C. elegans*, the *unc-47* gene is expressed in all 26 GABAergic neurons, including 19 D-type neurons of the ventral nerve cord and the postanal cell DVB (Figure 1A) [40]. We selected these cells because they are easy to recognize due to a characteristic morphology, and they reside close to the body surface, thus easing the quantification of expression. The endogenous pattern of *unc-47* is recapitulated when a reporter construct containing a 1.2 kb sequence immediately 5′ of the gene (we refer to it as a full-length promoter as it extends to the locus of the upstream gene) is used to drive green fluorescent protein (GFP) in *C. elegans* (Figure 1B). A construct containing a promoter of the same length of the *C. briggsae unc-47* ortholog is expressed in a qualitatively indistinguishable pattern in *C. briggsae* (Figure 1C). Indeed the *C. briggsae* promoter drives expression in the same neurons even in *C. elegans* (see below). These results suggest that expression patterns of *unc-47* orthologs have been conserved since their common ancestor and that the information required for driving proper expression is contained within ∼1.2 kb promoters upstream of the genes.

Because expression patterns of nematode *unc-47* orthologs are conserved, we investigated whether expression is mediated solely by conserved *cis*-regulatory elements. We aligned the *C. briggsae* sequence along with those of two other close relatives *C. brenneri* and *C. remanei*, to the *C. elegans unc-47* promoter. As reported previously [41], sequence conservation in this promoter is heavily biased to the most proximal ∼250 bp (Figure 1D, Figure S1, Table S1). We carried out extensive analyses which showed that little sequence conservation can be found distal to the ∼250 bp boundary (Figure S2, Table S1). This does not exclude the possibility that there exist short and conserved motifs in the distal promoter; they are simply below our level of detection. Some may exist and even be functional; nonetheless, the rates of sequence divergence are profoundly different between the proximal and the distal portions of this promoter.

If the conserved expression patterns result from solely the conserved portions of the *cis*-regulatory elements, then the proximal promoters of both *C. elegans* and *C. briggsae* should be sufficient to recapitulate the entire pattern. We therefore compared functions, in *C. elegans*, of both full-length and proximal promoters (Figure 1E) derived from *C. elegans* and *C. briggsae*. Strains bearing each of these four constructs exhibited qualitatively similar patterns of expression. However, we noticed that the proximal *C. briggsae* promoter was not robust – it drove both weak and inconsistent expression. In contrast, a robust promoter would express strongly and consistently, as do the full-length promoters. We next quantified and compared expression patterns driven by these promoters.

### Regulatory elements lacking sequence conservation are required for robust gene expression

The expression patterns driven by the proximal and full-length promoters from both species were qualitatively correct, that is, all cells that were expected to show reporter gene expression were GFP-positive in at least some of the examined animals. To obtain a precise measure of variability, we counted the number of D-type neurons that were expressing GFP in 200 individuals bearing each construct. We examined animals from multiple independent strains for each construct and found that overall inter-strain variance was modest for all constructs (data not shown). We conducted the counts in a blinded fashion to exclude the possibility of unconscious experimenter bias (see Methods). The results of these counts address the first aspect of robustness – consistency of expression pattern. We found that the full-length *C. elegans* promoter drove somewhat more consistent pattern than the proximal promoter (Figure 1F; Wilcoxon test, p = 3.2×10^−3^), and that the full-length promoters of *C. elegans* and *C. briggsae* were indistinguishable (p = 0.7). The *C. briggsae* proximal promoter was not expressed as consistently in D-type neurons as the full-length promoter (Figure 1G; Wilcoxon test, p = 3.4×10^−8^).

In a parallel approach we quantified the intensity of GFP fluorescence in DVB and D-type neurons. This allowed us to assess the second aspect of robust expression – consistency of relative expression levels from one cell type to another within an individual. Expression levels in D-type neurons and DVB were relatively similar in animals carrying the full-length promoter (note the mean ratio of one and a tight, normal scatter, Figure 1H). In contrast, individuals with the proximal promoter exhibited a significant increase in variance (Ansari-Bradley test, p = 1.6×10^−3^), despite a lower relative expression in DVB (Wilcoxon test, p = 1.8×10^−5^). We thus concluded that the *C. briggsae* proximal promoter directs less robust expression than the full-length promoter.

To ensure that the apparent decrease in robustness of the proximal promoter was not an artifact of using extrachromosomal arrays, we generated transgenic strains in which single-copy full-length or proximal promoters were integrated into the same genomic location. Whereas the absolute levels of expression were considerably lower for all integrated strains (20–400 fold), the shorter promoter was weaker than the full-length (4–6 fold) and significantly less consistent in its expression (Figure S3; Wilcoxon test, p = 1.9×10^−10^). Thus the shorter promoter was weaker and less consistently expressed regardless of whether it was tested as an integrated or extrachromosomal transgene. This concordance allowed us to utilize extrachromosomal transgenes for the remainder of this study, because integrated strains showed weak expression that was at the limit of detection.

**Figure 2:**
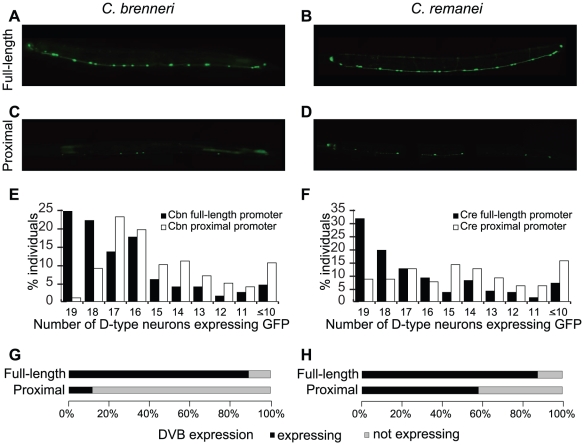
Promoters of *C. brenneri* and *C. remanei unc-47* require the distal sequences for consistent expression. GFP expression driven by full-length *C. brenneri* (A) and *C. remanei* (B) and proximal *C. brenneri* (C) and *C. remanei* (D) *unc-47* promoters in a *C. elegans* host. As in Figure 1F, 1G, 200 individuals bearing each transgene were counted, and the percentages of those individuals with the indicated number of D-type neurons expressing GFP is shown (E–F). (G–H) Presence/absence of GFP expression in the cell DVB in the same 200 individuals for each of the four promoters. Photographs of worms are composites of multiple exposures of the same individual that capture the full complement of D-type neurons, in all focal planes, expressing GFP.

It is formally possible that our observation of the decreased robustness of the proximal promoter compared to the full-length version was due to a peculiar nature of the *C. briggsae* regulatory sequence. We therefore tested orthologous *cis*-regulatory sequences of two additional species, *C. brenneri* and *C. remanei*, in *C. elegans*. Their full-length promoters drove GFP in a strong and consistent pattern, statistically indistinguishable from those of *C. elegans* and *C. briggsae* orthologs (Figure 2A, 2B). Both proximal promoters, truncated at the orthologous position at the boundary of conserved sequences around 250 bp, directed weaker (Figure 2C, 2D) and less robust (Figure 2E, 2F; Wilcoxon test, *C. brenneri* p = 1.2×10^−13^ and *C. remanei* p = 1.3×10^−13^) expression in D-type neurons. Expression of the proximal promoters was also less consistent in the tail neuron DVB (Figure 2G, 2H).

Our results suggest that the *cis*-regulatory elements of *unc-47* from the four examined nematodes have similar architectural properties – the proximal, highly conserved promoter is sufficient to deliver the qualitatively correct expression pattern, whereas the distal, nonconserved portion is required for consistent expression. It is important to note that this distal sequence is not alone sufficient to direct any expression in D-type neurons or DVB [41]. It therefore contributes to robustness via a mechanism different from that of recently described “shadow” enhancers [35], [36], each of which is sufficient to drive expression independently. Furthermore “shadow” enhancers are conserved, whereas the distal promoter of *unc-47* is not.

### Distal nonconserved promoter sequences are required to confer environmental robustness

**Figure 3:**
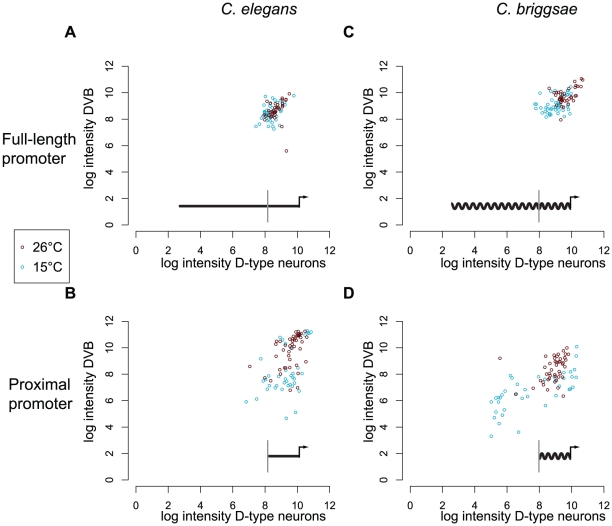
Only full-length promoters of *C. elegans* and *C. briggsae unc-47* can direct robust expression. Distribution of fluorescence intensity driven by *C. elegans* full-length (A) and proximal (B), and *C. briggsae* full-length (C) and proximal (D) promoters in *C. elegans* at two temperatures (red for 26°C and blue for 15°C). For each individual, the log intensity in D-type neurons is plotted against the log intensity in DVB. Individuals that did not show any fluorescence in DVB were excluded from analysis. Data for additional strains are given in Figure S4. Superimposed on each graph is a schematic of the construct used: a straight line represents *C. elegans* promoter of *unc-47*, a wavy line represents *C. briggsae* promoter of *unc-47*. The gray vertical bar indicates the 5′ boundary of the proximal promoter.

Distal promoters were required for stronger and more consistent expression, even when worms were reared under constant and nearly optimal growth conditions (20°C). We tested whether these sequences could also buffer against environmental challenges. We compared GFP expression levels directed by the full-length and proximal promoters in worms reared at a high temperature of 26°C and a low of 15°C. We measured the intensity of GFP-fluorescence in D-type neurons and DVB and observed several trends. First, expression levels driven by the full-length *C. elegans* promoter (Figure 3A) were more consistent than those driven by the proximal promoter (Figure 3B) at both the 26°C (Kolmogorov-Smirnov test, p = 2.9×10^−5^) and 15°C (p = 1.2×10^−6^). Second, the full-length promoter was comparably consistent in its expression at 26°C and 15°C (Figure 3A, Table S2). In contrast, consistency of expression of the proximal promoter differed dramatically between the two temperatures (Figure 3B, Table S2).

Similar results were observed for the *C. briggsae* promoters. The full-length promoter (Figure 3C) directed more consistent expression than the proximal promoter (Figure 3D) at both temperatures (Kolmogorov-Smirnov test, at 26°C p = 1.2×10^−2^, at 15°C p = 2.2×10^−14^). Temperature had a minor effect on the consistency of expression of the full-length promoter, but a more substantial effect on the proximal promoter (Table S2). We repeated measurements for multiple independent strains carrying full-length and proximal promoters from *C. elegans* and *C. briggsae* and observed concordant results (Figure S4, Table S2).

We concluded that full-length promoters are more robust to temperature stress, regardless of their species of origin (compare Figure 3A and 3C). Proximal promoters, primarily composed of conserved sequences, were significantly less robust, particularly after the cold treatment (Figure 3B and 3D). These results indicate that a robustness-conferring function is encoded in distal promoters in both species, and is thus conserved despite the lack of detectable sequence conservation.

### Distinct sequences in distal promoters can contribute to robust expression

**Figure 4:**
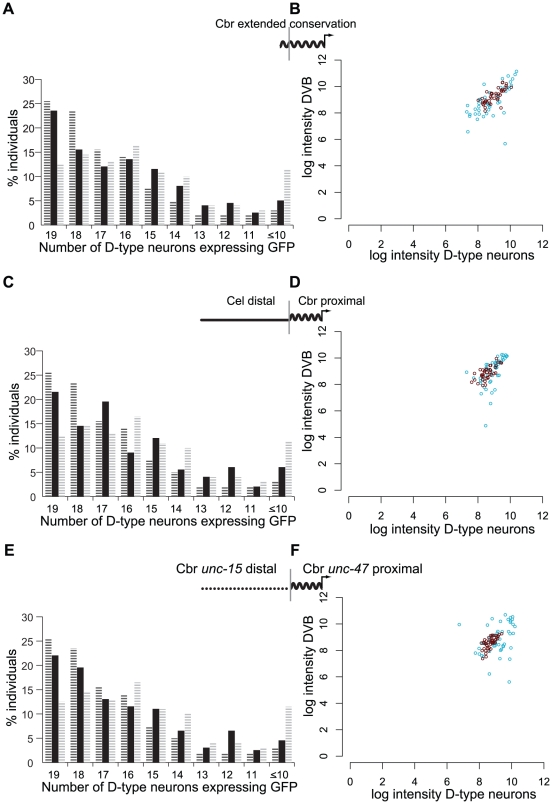
Different components of the distal promoter sequences regulate consistent expression and expression levels under temperature stress. (A) Percentage of 200 individuals expressing GFP in the indicated number of D-type neurons under control of *C. briggsae* promoter with extended conservation, shown in solid black bars compared to *C. briggsae* full-length (black hashed bars) and proximal (gray hashed bars) promoters. (B) Intensity of GFP expression in D-type neurons and the cell DVB for animals bearing the extended conservation promoter reared at 26°C (red) or 15°C (blue). (C) Percentage of 200 individuals expressing GFP in the indicated number of D-type neurons under control of chimeric promoter fusion of *C. elegans* distal *unc-47* promoter sequence and *C. briggsae* proximal promoter. For comparison, distributions for *C. briggsae* full-length and proximal promoters are shown in black and gray hashes, respectively. (D) Intensity of GFP in D-type neurons and the cell DVB for animals bearing the chimeric promoter reared at 26°C (red) or 15°C (blue). The chimeric promoter drives robust expression under temperature stress. (E) Percentage of 200 individuals expressing GFP in indicated number of D-type neurons from a chimeric promoter composed of distal *C. briggsae unc-15* sequence and the *C. briggsae unc-47* proximal promoter (black bars). For comparison, *C. briggsae unc-47* full-length and proximal promoters are shown in black and gray hashed bars, respectively. The *unc-15/unc-47* chimera is indistinguishable from the *C. briggsae* full-length promoter (Wilcoxon test p = 0.37), and it is significantly more consistent than the proximal promoter (Wilcoxon test p = 1.3×10^−5^). (F) Robustness of *unc-15/unc-47* chimeric promoter under temperature stress.

We dissected the distal promoters to determine which of their components were necessary for robust expression. The proximal promoters contain all of the densely arranged blocks of sequence conservation. Additionally, a pair of short motifs (8 and 6 bp) that is shared by all four examined nematodes is located approximately 50 bp distal to the boundary of greatest conservation (1). We considered the distal extent of these motifs to be the absolute boundary of the evolutionarily conserved promoter sequence, because in the remaining distal promoter there were no sequences longer than 10 bp that were shared by all four species. We tested a promoter encompassing all of this “extended conservation” for the ability to drive robust expression. It performed intermediately in terms of consistency of expression between the full-length *C. briggsae* promoter (Figure 4A; Wilcoxon test, p = 1.4×10^−2^) and the proximal promoter alone (p = 4.0×10^−3^). We next examined intensity of GFP expression in the D-type neurons and DVB in animals reared under temperature stress. At 15°C, although not at 26°C, this promoter produced more variable expression than the full-length *C. briggsae* promoter (compare Figure 4B and Figure 3C; Kolmogorov-Smirnov test, p = 5.2×10^−4^), but significantly less variable expression than the proximal promoter (compare Figure 4B and Figure 3D; p = 7.7×10^−5^). Therefore the two conserved motifs and the sequences that surround them contribute to, but do not entirely account for the robustness of the longer promoter.

Our results suggest that, despite substantial sequence divergence, distal promoters of *C. elegans* and *C. briggsae unc-47* confer robust expression to their respective proximal promoters (Figure 1F, 1G, Figure 3). To test whether distal promoters confer robustness in a species-specific manner, we asked whether the distal promoter of *C. elegans* could restore robust expression when fused to the proximal promoter of *C. briggsae*. We reasoned that if the distal and proximal sequence function as a unit and make up a single *cis*-regulatory element, the distal part of which has diverged considerably in its sequence, we should expect a chimeric construct not to rescue robustness. If, on the other hand, the proximal, highly conserved promoter and the distal promoter are two distinct functional units, they should be modular.

The *C. elegans*-distal-*C. briggsae*-proximal chimeric *unc-47* promoter drove expression with a consistency intermediate between the full-length and proximal promoters in terms of cell number (Figure 4C; Wilcoxon test, different from *C. briggsae* full-length p = 8.0×10^−3^; different from *C. briggsae* proximal p = 5.6×10^−3^). However, at both 15°C and 26°C this promoter was no more variable than the full-length *C. briggsae* construct (Figure 4D; Kolmogorov-Smirnov test, at 26°C p = 0.6, at 15°C p = 0.1), constituting a significant rescue of robustness relative to the proximal promoter alone (Kolmogorov-Smirnov test, at 26°C p = 4.6×10^−4^, at 15°C p = 1.2×10^−10^). Because much, although perhaps not all, of the robustness of expression can be rescued by this chimeric construct, we conclude that the proximal and distal sequences encode distinct and separable regulatory functions. Multiple chimeric and “extended conservation” constructs were consistent with these results (Figure S5, Table S2).

The robustness function of the distal element must have much less stringent sequence requirements than the proximal promoter, because distal sequences have diverged considerably but maintain this function. We next tested whether another genomic fragment lacking detectable sequence similarity to the distal *unc-47* sequences could confer robustness of expression. We selected an approximately 1.3 kb fragment upstream of *unc-15* because it does not share significant similarity with the *C. briggsae unc-47* distal promoter (Figure S2). Furthermore, *unc-15* encodes a paramyosin ortholog that is expressed in muscles [42], and thus is not expressed in any of the same cells as *unc-47*. The overall length of this sequence is comparable, however, and it is also an intergenic sequence as poorly conserved between *C. elegans* and *C. briggsae* as is the distal portion of the *unc-47* promoter (data not shown).

We were surprised to find that the chimeric promoter containing this distal *C. briggsae unc-15* sequence fused to the proximal *C. briggsae unc-47* promoter displayed robust expression as consistent as the full-length *C. briggsae unc-47* promoter in terms of cell number (Figure 4E; Wilcoxon test, p = 0.37). We observed markedly improved consistency of the expression pattern over the *C. briggsae* proximal promoter alone (Figure 4F; difference from proximal promoter, Wilcoxon test, p = 1.3×10^−5^). At 26°C this promoter drove as consistent expression as the full-length *C. briggsae* promoter (Kolmogorov-Smirnov test, at 26°C p = 0.1; compare Figure 4F, Figure S5 and Figure 3C). Whereas at 15°C, it was less consistent than the full-length *C. briggsae* promoter (Kolmogorov-Smirnov test, at 15°C p = 2.4×10^−4^), it was significantly more consistent than the proximal promoter at both temperatures (compare Figure 4F, Figure S5 and Figure 3D; Kolmogorov-Smirnov test, at 15°C p = 4.1×10^−7^, at 26°C p = 1.4×10^−5^).

Next, we tested whether another non-conserved intergenic sequence, from upstream of the *C. briggsae* promoter of gene *unc-25* could rescue robustness of the proximal *C. briggsae* promoter of *unc-47*. Unlike *unc-15*, *unc-25* is co-expressed with *unc-47* [43], yet it shares no detectable sequence similarity within promoter elements (data not shown). It did indeed show substantially increased robustness of expression, comparable to the full-length promoter (Figure S6; indistinguishable from *C. briggsae* full-length Wilcoxon test, p = 0.4; different from *C. briggsae* proximal Wilcoxon test, p = 1.3×10^−5^). These results show that unrelated intergenic sequences are capable of conferring robust expression on a proximal promoter that directs the pattern.

### Sequences that confer robust expression are AT-enriched

To understand why such different sequences were able to restore robustness of expression of the proximal *C. briggsae unc-47* promoter, we examined them for general features they might have in common. Specifically, we calculated nucleotide frequencies in the distal *unc-47*, *unc-15* and *unc-25* promoters, and compared them to those of the 1.1 kb of vector DNA sequence that lies distal to all of the inserted promoters. Since this vector sequence, when it lies directly upstream of the proximal promoter, is not able to confer robustness, we sought out features that are shared by distal promoters but not the vector sequence.

**Figure 5:**
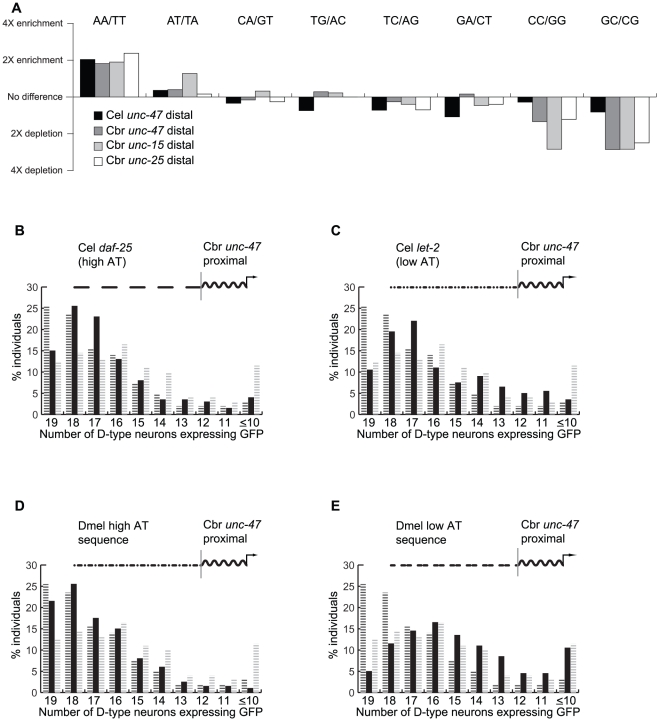
Promoter regions that confer robustness are enriched for nucleosome-depleted sequences. (A) Enrichment/depletion of dinucleotides in the distal promoters of *unc-15*, *unc-25* and *unc-47* genes relative to the sequence of the pPD95.75 vector (log scale). Percentage of 200 individuals expressing GFP in indicated number of D-type neurons from a chimeric promoter composed of AT-rich sequence from the *C. elegans daf-25* locus (B), AT-poor sequence from the *C. elegans let-2* locus (C), AT-rich sequence from the *D. melanogaster ChAT* locus (D), and AT-poor sequence from the *D. melanogaster* CG8394.2 locus (E), fused upstream of the *C. briggsae unc-47* proximal promoter. For comparison, *C. briggsae unc-47* full-length and proximal promoters are shown in black and gray hashed bars, respectively.

Dinucleotide frequencies differ dramatically between distal *unc-47*, *unc-15* and *unc-25* promoter sequences and the upstream vector sequence. There is systematic enrichment for two dinucleotide classes, relative to the vector sequence, and a depletion of two other dinucleotide classes (Figure 5A). While there are between-sequence enrichment differences, the overall biases towards the AA/TT dinucleotides and away from the GC/CG dinucleotides is consistent among all sequences that confer robustness.

This analysis suggests a simple hypothesis, namely that AT-enriched sequences (more specifically those enriched for AA/TT dinucleotides) should promote robust expression, whereas sequences depleted for these dinucleotides and enriched for GC/CG pairs (and to some extent CC/GG pairs) should not. To test this prediction, we subdivided the genome of *C. elegans* into 1 kb fragments, matching in size the previously tested distal sequences, and computed the extent of their AT-enrichment. A sequence located downstream of the *daf-25* locus is enriched for AA/TT dinucleotides to an extent similar to distal promoters of *unc-47*, *unc-15* and *unc-25*. This 1 kb fragment, when placed upstream of the proximal promoter of *C. briggsae unc-47*, was able to confer robustness similarly to the distal *unc-47* promoter (Figure 5B; indistinguishable from *C. briggsae* full-length Wilcoxon test, p = 0.09; different from *C. briggsae* proximal Wilcoxon test, p = 1.9×10^−5^). In contrast, a 1 kb AT-depleted sequence from the *let-2* locus was unable to rescue robustness (Figure 5C; different from *C. briggsae* full-length Wilcoxon test, p = 1.1×10^−5^; indistinguishable from *C. briggsae* proximal Wilcoxon test, p = 0.14). Furthermore, the construct containing the *daf-25* sequence drove a more consistent expression than the one containing the *let-2* sequence (Wilcoxon test, p = 4×10^−3^).

To ensure that the ability to rescue expression robustness is not restricted to AT-enriched sequences from nematode genomes, we tested whether sequences from distantly related species can perform this function. We segmented the genome of *D. melanogaster* into 1 kb fragments and selected one AT-enriched and one AT-depleted sequence using the same criteria as were applied to the fragments from the *C. elegans* genome. As predicted, a construct carrying the AT-enriched sequence drove substantially more robust expression than the proximal promoter alone (Figure 5D; indistinguishable from *C. briggsae* full-length Wilcoxon test, p = 0.2; different from *C. briggsae* proximal Wilcoxon test, p = 1.5×10^−5^). A construct carrying the AT-depleted sequence was no more robust than the proximal promoter alone (Figure 5E; different from *C. briggsae* full-length Wilcoxon test, p = 3.7×10^−15^; indistinguishable from *C. briggsae* proximal Wilcoxon test, p = 0.04).

Together these results suggest three important conclusions. First, AT-enrichment of a sequence can predict its ability to confer robustness of expression. Second, because two different AT-depleted sequences were not able to improve consistency of transgene expression, it is unlikely that robustness results from simply separating the proximal promoter from unknown repressive effects of the vector sequence. Sequence composition must play a critical role. Third, because multiple unrelated nematode sequences and an AT-enriched Drosophila sequence conferred robust expression, it is unlikely that short, gene- or species-specific motifs play a major role in improving consistency of expression. Our data imply that the mechanism responsible for conferring expression robustness relies on the overall nucleotide composition of promoters rather then on specific sequence motifs.

## Discussion

Our results suggest that promoters of Caenorhabditis *unc-47* orthologs are organized into two domains that are markedly distinct in functions and evolutionary dynamics. Whereas proximal promoters are highly conserved and are sufficient to direct the appropriate spatial expression pattern, the distal sequences diverge rapidly and their primary function is to confer robustness of expression. The distal sequences within promoters of *unc-47* are not capable of directing expression patterns on their own [41] and must therefore confer robustness via a mechanism distinct from redundant and evolutionarily conserved “shadow” enhancers [35], [36].

The shared nucleotide composition (Figure 5A) of the four sequences that promote robust expression – distal promoters of *C. elegans* and *C. briggsae unc-47* as well as upstream regions of two unrelated genes, *unc-15* and *unc-25* – hints at a potential mechanism of action. Overall sequence composition plays a large role in establishing chromatin states throughout the genome [44]. In particular, AT-rich sequences tend to be associated with nucleosome-poor regions, although multiple factors determine whether DNA is bound to nucleosomes. Recent studies suggest that sequence-composition codes that displace nucleosomes may be common in active metazoan promoters [45], [46]. Intriguingly, the genomic sequence precisely corresponding to the distal, nonconserved portion of the *C. elegans unc-47* promoter is depleted of nucleosomes [47] (Figure S7).

Trinucleotide frequencies are a better predictor of nucleosome positioning than dinucleotides [47]. The robustness-conferring sequences are two-fold enriched for trinucleotides that are preferentially found in nucleosome-depleted regions of the *C. elegans* genome, far more so than the conserved proximal promoters (Figure S7). Nucleosome occupancy can differ even in evolutionarily conserved promoters [46], [48], [49], still similar levels of enrichment for nucleosome-depleted trinucleotides were seen in the distal *unc-47* promoters of *C. brenneri* and *C. remanei* (Figure S7). All sequences that confer robustness bear a signature consistent with nucleosome depletion, and the *C. elegans* sequences were shown to be depleted of nucleosomes (Figure S7). The AT-poor *let-2* locus, on the other hand, is enriched for nucleosomes, and other sequences which are unable to improve consistency of expression, show a trinucleotide signature of nucleosome enrichment (Figure S7). We therefore hypothesize that open chromatin may promote robust expression.

We favor the hypothesis that the robustness function is executed by configuring chromatin in an accessible state for other factors to bind the promoter sequence. This hypothesis is consistent with the finding that variability of gene expression may be encoded in nucleosome-positioning sequences [50], and that chromatin regulators may contribute to environmental canalization [51]. Whether this mechanism of robustness arises as a byproduct of other forces that shape nucleotide composition of intergenic sequences, or whether it is directly selected upon, it has been conserved at the *unc-47* locus.

We propose a simple scenario to account for the different evolutionary rates between the distal and proximal portions of the *unc-47* promoter. The proximal promoter is responsible for directing the expression pattern because it contains numerous transcription factor binding sites. It appears that in the context of the proximal promoter most substitutions are deleterious and thus it evolves relatively slowly. The distal promoter, on the other hand, evolves at a considerably faster rate. Noting that the ability to confer robustness is conserved between distal promoters of *unc-47* orthologs, we infer that it is maintained by selection that does not require maintenance of specific sequence identity. Indeed, unrelated sequences from the *C. elegans unc-15*, *unc-25*, and *daf-25* loci and even an AT-rich sequence from *D. melanogaster* can rescue robustness of expression. Thus the distal promoters appear to be under a simpler constraint – they are only required to maintain a certain nucleotide composition, for instance that which is consistent with nucleosome depletion, to confer robustness of gene expression. Sequences that satisfy this requirement are quite degenerate, so the element tolerates a relatively high rate of sequence turnover, while retaining functional conservation. This hypothesis is consistent with a report of selection on sequence composition that encodes nucleosome organization in yeast [52]. We consider the distal promoter of the *unc-47* gene to be an example of a weakly constrained functional sequence [53]. Such low constraint allows developmental systems drift [54], in which conserved molecular functions are mediated by divergent genetic systems.

## Methods

### Constructs and strains

To generate reporter constructs, promoter sequences were PCR amplified from genomic DNA and cloned upstream of GFP into pPD95.75. In all cases, reverse primers overlapped the start codon of the *unc-47* ortholog. Prior to injections, constructs were sequenced to ensure accuracy. Precise boundaries of full-length, extended conservation and proximal constructs are given in Figure S1. To generate strains carrying extrachromosomal arrays, we injected a mixture (5 ng/µL promoter::GFP plasmid, 5 ng/µL *pha-1* rescue construct, 100 ng/µL salmon sperm DNA) into *C. elegans pha-1* (e2123) strain [55]. Transformants were selected at 25°C. The *C. briggsae* strains carrying Cbr promoter *unc-47*::GFP were produced by injecting a mixture (5 ng/µL promoter::GFP plasmid and 100 ng/µL salmon sperm DNA) into AF16 strain. Single copy integrated strains were generated following an established protocol [56]. Copy number of inserts was verified through quantitative PCR of GFP (normalized to genomic *unc-47*).

### Counting the number of expressing cells

Mixed-stage populations of *C. elegans* carrying transgenes were grown at 20°C with abundant food and young adult- or L4-stage worms were selected. These were immobilized on agar slides with 100 mM NaN_3_ in M9 buffer. The slides were examined on a Leica DM5000B compound microscope under 400× magnification. Each worm was positioned such that the ventral nerve cord with its D-type neurons could be seen clearly, and the number of cell bodies expressing GFP were counted manually. Worms without any visible GFP expression were assumed to have lost the transgene. For each construct studied, multiple independent transgenic lines were generated, and final counts of 100–200 individuals (see figure legends/text for details) were derived from a mixture of these lines (inter-line variance is generally low). To mitigate against experimenter bias census counts were taken in a blinded fashion. Individual strains were coded by one investigator to obscure their identity. Another investigator then examined 100 individuals of each of these strains. Once all counting was finished, strain identities were revealed and data were analyzed.

### Fluorescence measurements and temperature stress experiments

Intensity of GFP expression in individual cells was measured on a Leica DM5000B compound scope fitted with a Qimaging Retiga2000 camera. Images of cells were outlined in imageJ, average intensity was measured and the background subtracted. Multiple strains carrying the same transgene were examined throughout and tested for concordance.

For integrated strains we used 125 ms exposure, 100% excitation. Pictures of 7 cells (DD1, VD1, VD2, DD3, VD6, VD13, DVB) were taken. For each strain and treatment (15°C, 20°C, 26°C) 25 L4-staged worms were measured. For temperature stress experiments (these were conducted on strains carrying extrachromosomal arrays) worms were reared at 15°C or 26°C for at least two generations. Then 50 L4 individuals were mounted for each treatment and strain and intensity of GFP was measured (125 ms exposure, variable excitation) for D-type neurons (average values recorded) and DVB.

### Statistical analysis

All statistical analyses were performed in R. In all cases, the logarithm of measured GFP intensity was used. Wilcoxon test was used to assess consistency of the number of cells expressing different constructs. To assess the amount of scatter in fluorescence measurements (data reported in Figure 3, Figure 4, Figures S4 and S5, and in Table S2), we computed geometric distances between all data points for a particular strain/treatment and the mean of that strain/treatment. To test whether distributions of distances derived in such a way were significantly different for different strains/treatments, we conducted Kolmogorov-Smirnov tests. We used Ansari-Bradley test to determine whether the relative DVB fluorescence was more variable for proximal compared to full-length promoters.

## Supporting Information

Figure S1 Annotated alignment of *unc-47* upstream sequences from four nematode species.
(PDF)

Figure S2 Sequence conservation of *unc-47* is biased to the proximal promoter.
(PDF)

Figure S3 Integrated and extrachromosomal transgenes show corresponding effect of promoter length on pattern consistency.
(PDF)

Figure S4 Multiple independent full-length and proximal strains are consistent in their robustness.
(PDF)

Figure S5 Multiple independent chimeric and deleted strains are consistent in their robustness.
(PDF)

Figure S6 The *C. briggsae unc-25* distal promoter confers robustness of expression pattern.
(PDF)

Figure S7 Nucleosome occupancy in robustness-conferring sequences.
(PDF)

Table S1 Conservation of proximal promoters and distal sequence among four nematode species.
(PDF)

Table S2 Comparisons of variation of all strains at 15°C and 26°C.
(PDF)
