# Supplementary material for: Distinct Functional Constraints Partition Sequence Conservation in a cis-Regulatory Element
Source: PLoS Genet. 2011 Jun 2;7(6):e1002095. doi: 10.1371/journal.pgen.1002095 (PMC3107193; doi:10.1371/journal.pgen.1002095)
Supplement: Figure S1 — Annotated alignment of unc-47 upstream sequences from four nematode species. (PDF) [file pgen.1002095.s001.pdf]

Figure S1. Annotated alignment of *unc-47* upstream sequences from 4 nematode species.

```
       ....|....| ....|....| ....|....| ....|....| ....|....| ....|....| ....|....| ....|....| ....|....| ....|....|
                10         20         30         40         50         60         70         80         90        100
C.ele  ~~~~~~~~~~ ~~~~~~~~~~ ~~~~~~~~~~ ~~~~~~~~~~ ~~~~~~~~~~ ~~~~~~~~~~ ~~~~~~~~~~ ~~~~~ATCCC GGAACAGTCG AAAGTCGG--
C.bri  ~~~~~~~~~~ ~~~~~~~~~~ ~~~~~~~~~~ ~~~~~~~~~~ ~~~~~~~~~~ ~~~~~~~~~~ ~~~~~~~~~~ ~~~~~~~~~~ ~~~~~~~~~~ ~~~~~~~GGG
C.rem  AAGCTTGGGA AAGATCGGAA AAGATGATAG GATATATTGA AAAGGATCTG TTTCGTTAAA GCAACTCATC CCAGTAAATT GTGAACTTTT CAA-------
C.bre  ~~~~~~~~~~ ~~~~~~~~~~ ~~~~~~~~~~ ~~~~~~~~~~ ~~~~~~~~~~ ~~~~~~~~~~ ~~~~~~~~~~ ~~~~~~~~~~ ~~~~~~~~~~ ~~~~~~~~~~
```

start of full-length promoter sequences in red

```
       ....|....| ....|....| ....|....| ....|....| ....|....| ....|....| ....|....| ....|....| ....|....| ....|....|
               110        120        130        140        150        160        170        180        190        200
C.ele  -TGGCAAGCG CCGAACTGCT GACGGTCTAA CCGGGG---- -CACAAATCA GGGGTGAGCG GCAAACGATT TTTCCGGCAA AT--CGGCAA ATCGGCAAAT
C.bri  ATTCGGAGAA CAGTAACTCA AAAGCTCGAA ATATGATTCT TCAACTTTTC AAAGTTTTCT TTGATATACA CAGGTAGAGG GGCATTAAAC TGCTAAATGA
C.rem  -TGATAAACC GTGCAAC-TG ATCGAGTGAA A-ATGG---- -CATAG---T AAAGGATACA GTAATCTCCA ACAACAAAAA AAAATCAGAA ATCCAGGAAT
C.bre  ~~~~~~~~~~ ~~~~~~~~~~ ~~~~~~~~~~ ~~~~~~~~~~ ~~~~~~~~~~ ~~~~~~~~~~ ~~~~~~~~~~ ~~~~~~~~~~ ~~~~~~~~~~ ~~~~~~~~~~

       ....|....| ....|....| ....|....| ....|....| ....|....| ....|....| ....|....| ....|....| ....|....| ....|....|
               210        220        230        240        250        260        270        280        290        300
C.ele  TGCCAATATT GAAATACCCG GCAAATCGGT AAATAGCCGG AA--TTGAAA ATTTCCGGCA AACTGGTA-- -AACCGCAAA TTGCTGATTT G-----CC--
C.bri  CAGCCAAAGA GGAGCAAAAT GGCGAATGAC TGCTAGTTGG AAGCCGAAGG AGGACCGATA GACTGTCAAC GGACTGTGGA CGGACAACCG GAACCTCTTG
C.rem  CAATATTAAG AAAATAACAT AT--AATCCC AGTT--TTCC TA-CCGTATA TTCACCATAA TAATGAC--- --ATCATTGA GTTTCTACGA GAAACACT--
C.bre  ~~~~~~~~~~ ~~~~~~~~~~ ~~~~~~~~~~ ~~~~~~~~~~ ~~~~~~~~~~ ~~~~~~~~~~ ~~~~~~~~~~ ~~~~~~~~~~ ~~~~~~~~~~ ~~~~~~~~~~

       ....|....| ....|....| ....|....| ....|....| ....|....| ....|....| ....|....| ....|....| ....|....| ....|....|
               310        320        330        340        350        360        370        380        390        400
C.ele  -GAATTTGCC GGGAAGACGG CAATTG---- ----CCAAAC ATATTC---- ---------- --GGCAAATT GTGGTTTT-G CACTTTTTGG AAATTTCAGA
C.bri  GAAAACCGAC TTGAAAGTTT TGATTGAAAA TCATCCAGAA AAGATTATGT TATGCTTCCA CTAGATTATT CTGATGATAG AAACTACTTT TTCTCACATA
C.rem  GAAAGCTGAA G-TCAAAATT TTATTAAG-- --AGCCATAA AGATT----- ---------- --AAAAAAGT ATTTTGTCAG GAGTTTGTGT --CTTACAAA
C.bre  ~~~~~~~~~~ ~~~~~~~~~~ ~~~~~~~~~~ ~~~~~~~~~~ ~~~~~~~~~~ ~~~~~~~~~~ ~~CGCATGCT CCAGACTTAG GAATATATGG TACTCATCTG

       ....|....| ....|....| ....|....| ....|....| ....|....| ....|....| ....|....| ....|....| ....|....| ....|....|
               410        420        430        440        450        460        470        480        490        500
```

```
C.ele   A-----TTTC AATCGGCAAA TTGT------ ----GCACAT -----CCTAT GAATTTTCCT ACATCTATTT TGAAAAGTAA GC-----AAA TTCTATGAAA
C.bri   AGACAACCTC AAAAAGTATA TTGTTGAACG ACGGCTTGGT GACAGTCGAA AGGCACTGAC TAGTCGATTA CCGGTGGTTG GTGGACCAAA TTTCTGGAGA
C.rem   A------CTC AGAAA---AA TTTT-----G TTTTTTAATT -----TCTAA AAAATTTAAT TGG---ATCT TGAGTTCTCA GT----CGAA CTTA-AAGAA
C.bre   G------TGC AAATG---TA TTCCTATCAG ATCTCCAGAT -----CAGAG AAGTTTCGAA TGC---ATCA AGACTTTTCA CT----TACA GTTC-GGATA

        ....|....| ....|....| ....|....| ....|....| ....|....| ....|....| ....|....| ....|....| ....|....| ....|....|
               510        520        530        540        550        560        570        580        590        600
C.ele   ATATCTAAAG AAAAATGGAA AAAATTT--- --TCA----- ---AAAAGGC ACA----GTT TTAAGTGTTT CC----GTCT A---ATAAAA AAATCCCCCT
C.bri   ATGGAACATT TTGTCAAAAA TTACTTTTGG AATTATCACA CTATAAAAGT CAAAGAGGAC TCTGGGAATC TTCAAAATTT ATTATTAGAA GTATGCGGAC
C.rem   CTGGTACATT TGAGG--AAG CGAGACC--- -ATTG----- ---GAAAGGA GTA---TGAG CCACGTCATT ---AAATTGT G---GTAAGA -TACTTACTC
C.bre   TCATTACAGG ACAAGTTGAA CGAATCC--- -TCAGTCTCA CACGGAAGGT GTAGTCCAAT TGATATCATC CA-AAACCGT ATTTGTAGTG -AAACTAAGT

        ....|....| ....|....| ....|....| ....|....| ....|....| ....|....| ....|....| ....|....| ....|....| ....|....|
               610        620        630        640        650        660        670        680        690        700
C.ele   AAACACTTCC GGC--AAATT GATGTTCGGC AAATGGCAAA TC-------- GGAAACTTGC CGAAAAT--- ---------- TACAGTTTCC GGTAA-----
C.bri   AAAAATGACC CATTTATATT CAGAAGAAAT CTCGAAAACG TTTTTTTTTT GAAAACCAAG AGAAGAATGC GCCTGAACAG TGATGCTTAT CGAGGTAATA
C.rem   AATGAAGATT C----ACATC CAAAAACTGA TCACAAGGCG TA-------- GAAAGAACTG TGAACTG--- ---------- TAATTCTTTC GGACG-----
C.bre   AGAGAAGTTT CG---AGATC TCGTTGCCAT CCATAAAAAA AC-------- GAAAACGCTG TCATGTAAAT CCGGGA---- TGCTGCTTGC AGACATAAAT

        ....|....| ....|....| ....|....| ....|....| ....|....| ....|....| ....|....| ....|....| ....|....| ....|....|
               710        720        730        740        750        760        770        780        790        800
C.ele   -ATCGGCAAA CCGGCAAACT --------GC CTGAATTGAA AAGTTCCGTC AAATCGGCAA ACCGACAACA CCCCTGGCAC AAATGATGGA CATACTGAGG
C.bri   TAAAGACTTC AGGACTTAAC AATAAAACAA ATGCGTCCAG GAATTTTTGA AGCGGGTCTA GCCAATGTTT TTTTTCTTTT TCAAAATCCC TTTCTGTGAA
C.rem   -ATTGGATAC TAG-CGGAAT ---------- ACTC-T--AC AAAT-----A ATGAG---AA ACCAAGACCT GATTTCACCT GAAAAGCTCT TCTCCGTGAA
C.bre   AATCTATTTT TAA-CAGATT TGAGAGTGGA ATTCGT--AA GAAT-----C AGGAGCGAAA ATTAATGACA AATGACAGGT AGTGAGATGG AAAACTAGGA

        ....|....| ....|....| ....|....| ....|....| ....|....| ....|....| ....|....| ....|....| ....|....| ....|....|
               810        820        830        840        850        860        870        880        890        900
C.ele   CAATTTGCCG GT-------- --------TT TCCAATTGCA GGAAATTTT- ---CAATTCC GGCAGTGTGC CGA------- TTTGCCGGAA ATTTTAATTC
C.bri   AAATGATGCC GTGAGATGAA GTCCCAAATT TAGAAAAGTT TCAAACATTG AAACAACACA TTTGAGGAAT AGGCTCTTCT TATTTTAGTA GCCAAGCAAC
C.rem   GAAA------ CT-------- ---------- ---AAAAACA ACTGTCTT-- ---CACTTGA AATGAAG--- ---------- -----TGAAA ACAAAAAAGA
C.bre   GAAGGTTCCT CT-------- ---------- ---CAAAGTT TTGACCTT-- ---CAGTCCA ACTGAAGAGT ---------T TTCGATGGAA GTTGAAACAA
```

```
         ....|....| ....|....| ....|....| ....|....| ....|....| ....|....| ....|....| ....|....| ....|....| ....|....|
                910        920        930        940        950        960        970        980        990
1000
C.ele    AGGCAAATTG CCGATTTCCC GATTTCCCGA TTTGCCGGAA AAAATCGTTT GCCGCCCACC -------CCT ---------- ---------- --GGGT----
C.bri    AAACGAATTT CCATGCAACC CATCCGTAGA CATCAAAACG ATGCTCATCT CACAGTCGTC ACGTTTTTCT GAGAATGAAG AGAAACAAAA AAGGGGTTGG
C.rem    AGACGGA--- --AGAAAATC CAATTGAAAA CA--TAGG-- ----TTTACT CAAATTCACC --------CC ---------- ---------- --GAGA----
C.bre    AAAAGGA--- --GAAAAAGT GAATAGAAAA AAAGTGAA-- ----TTACCC CTCATTCAAT --------CT TTTGAAGAAA AAAGAACAGA ATGAGA----
```

```
         ....|....| ....|....| ....|....| ....|....| ....|....| ....|....| ....|....| ....|....| ....|....| ....|....|
                1010       1020       1030       1040       1050       1060       1070       1080       1090
1100
C.ele    --CTGAACCT TGATTGTTAC AAAACATT-T TTAGCTCTTT GGAGAAATAA AATGAATCTC GTAAAATTT- -AATTGACGA GGACGATATT AGCT----GT
C.bri    AAAGAAATCG AATAGGAAAT AGGGGATTAC TCAATTCCAA TCCCAACCGA ATCTCATTTC AAAAAACTCT CAACCAACTA ATCCCACATT GCCTCGAGAT
C.rem    AAATGTATTT AATTTCGATT TGAAAACCCC TCAGAATTTG AATTTTCTCA AGAAAAAGTC GGTGAATTTT CTAATGGACT GTACGA-ATG ACAT-----T
C.bre    AAAAGGATCT AATTTCAGAT GGTTCCTTTT TCAAAAGACT CCTAATCTAA TTCTTCTTTT AATAGTTTTC TTAATGGACT GTACAA-TTT CCCTCTGTGT
```

```
         ....|....| ....|....| ....|....| ....|....| ....|....| ....|....| ....|....| ....|....| ....|....| ....|....|
                1110       1120       1130       1140       1150       1160       1170       1180       1190
1200
C.ele    CTCTTT---- AGACCAAATT CAGAAAAAAA GAAAGAA--- ------TACT TCC~~~~~~~ ~~~~~~~~~~ ~~~~~~~~CA AATTTCCGG~ ~~~~~~~TCC
C.bri    TCTTTTTTCT GTAATGTGTT TCTGAATGGA CTGTAAGATT GATGTTCTCA TTTCCTTTTT CTCTAAAAAA CCAGCCTTCA AATTTCCGGA GCACAAGTCC
C.rem    TCCCCT---- -------GTT C----AAAAG CAATTA---- ------CCCA TTC---T--- ---------CA TTTTTCTCCA AATTTCCGGA --ATTGGTCC
C.bre    TCTTCC---- -------ATT CCATAAAAAA CCATTA---- ------CCCA TTC---TTTT AACAAGCCCA TTTTTCTTTG AATTTCCGTC --ATCGGTCC
```

start of *C. briggsae* extended conservation promoter in blue **distal blocks of conservation in yellow**

```
         ....|....| ....|....| ....|....| ....|....| ....|....| ....|....| ....|....| ....|....| ....|....| ....|....|
                1210       1220       1230       1240       1250       1260       1270       1280       1290
1300
C.ele    CTCTCTCGTT TTTTTTGCCA ATAAACTCAC TATAGTCGCT GGTTCCCCCC TATTCACATT TATTCTACCA ATCCATCAGT GG-------- --AACCAGA-
C.bri    CTCAACCCCA ATAGGTTCGC TCCTTTCCTC TTTT~~~~~~ ~~~~~~~~~~ ~~~CCTTATT CATTCCACTC ATCCATCAGG GAACCAAAAA GAAAACGAAT
C.rem    CTCTCCAT-- --~~~~~~~~ ~~AAAGTCTT CCCA----TT CATT~~~~~~ ~~~CCTT-TT CATTCCACTC ATCCATCAGT GGT------- GGAACCAAA-
C.bre    ~TCCCAT--- ---AAACTCG CTATAGTCCC TCTTT~~~~~ ~~~~~~~~~~ ~~~TCTT-TT CATTCCACTC ATCCATCAGT GGT------- GGAACCAAA-
```

start of proximal promoter sequences in gray

```
         ....|....| ....|....| ....|....| ....|....| ....|....| ....|....| ....|....| ....|....| ....|....| ....|....|
```

```
                1310       1320       1330       1340       1350       1360       1370       1380       1390
1400
C.ele   AAAAAGAAGA GCCTTTCGGT TTGGAGAGTA GGGTCTAATA ATCCCCCGTG CTCTTCAAAT CATTGTGCCA ACACACAGAC ACACTTTATG TGTGCTCACA
C.bri   AAGAAGAAGA GTCTTC---- --GGAGAAGA GCGTCTAATA ATCCCTG--- --CTTCAAAT CATTGTGCCA ACACA~~GAC ACACTTTATG GG~~CCAGAA
C.rem   ---AAGAAGA GCCTC----- -------GGA GCGTCTAATA ATCCCTG--- --CTTCAAAT CATTGTGCCA ACACA~~~~C ACACTTTATG TG--CCCA--
C.bre   ---AAGAAGA GCCTC----- -------GGA GCGTCTAATA ATCCCTG--- --CTTCAAAT CATTGTGCCA ACACA~~~~C ACACTTTATG TGTGCCCA--


        ....|....| ....|....| ....|....| ....|....| ....|....| ....|....| ....|....| ....|....| ....|....| ....|....|
                1410       1420       1430       1440       1450       1460       1470       1480       1490
1500
C.ele   CACACACGCT ATTTGAAGAG CGAAGACGAC GAC------- ---------G ACGCATTCAG A-GCTCTTTT CCACGAAATT TGCTCCATCT TTCCACAATC
C.bri   C---CACGCT ATTTGAAGAG CAACGACGAC GAT-GACG-- AGCGCCCAAG AGGTCTCCAG A-GCT-CTTT TCACAAATTC ----TCTTCT TTCAA-AACC
C.rem   ----CACGCT ATTTGAAGAG CGAAGAAGAC GACCGA---T GACGACCTCG ACGTCTTCAG ACGCT-CTTT TCAAAAAATT ----CTTTCT TTTCA-AATC
C.bre   ----CACGCT ATTTGAAGAG CGAAGAAGAC GTCCGACGAT GACGACGACG ACGTCTTCAG ACCCTTCTTT TCACGAATTT ----CTTATT TTTCG-AATT


        ....|....| ....|....| ....|....| ....|....| ....|..
                1510       1520       1530       1540
C.ele   TGTCTTTCCT GTGAGACGAC AGCGTCACAT TTATTTCATT ACAGATG
C.bri   GGTGGTTCCT TTCAAGT--- --TTGT--GT TTCCT-TACA G-ACATG
C.rem   GGTCTTCCTG CT-ACAT--- --TT----AT TTCGT-TACA GAAGATG
C.bre   TGTCTTACTT CTCATAC--- --CTCTCAAT TTCGTATTTT ACAGATG
```

C.ele=*C. elegans*; C.bri=*C. briggsae*; C.rem=*C. remanei*; C.bre=*C. brenneri*. All genomic sequences are available on Wormbase (http://wormbase.org/). Alignment was carried out using BioEdit (Hall, 1999) ClustalW Multiple Alignment function and was edited manually. All sequences terminate with the start codon of the *unc-47* ortholog.

1. Hall, T.A. (1999) BioEdit: a user-friendly biological sequence alignment editor and analysis program for Windows 95/98/NT. Nucl. Acids. Symp. Ser. 41:95-98.
