# Supplementary material for: Distinct Functional Constraints Partition Sequence Conservation in a cis-Regulatory Element
Source: PLoS Genet. 2011 Jun 2;7(6):e1002095. doi: 10.1371/journal.pgen.1002095 (PMC3107193; doi:10.1371/journal.pgen.1002095)
Supplement: Figure S2 — Sequence conservation of unc-47 is biased to the proximal promoter. (PDF) [file pgen.1002095.s002.pdf]

Figure S2. Sequence conservation of *unc-47* is biased to the proximal promoter

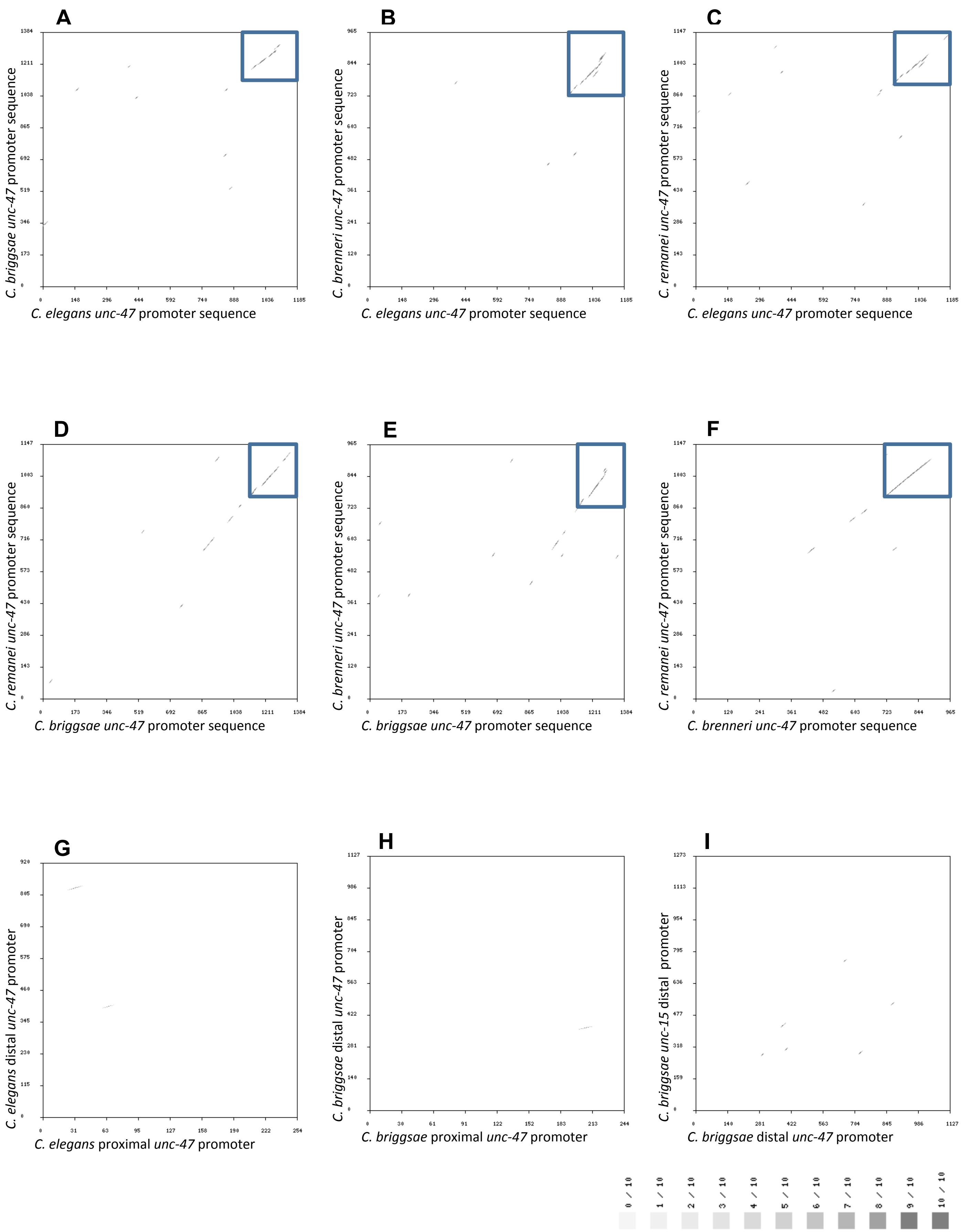

Figure S2. Dotplots showing matches of *unc-47* upstream sequence 10 bp or longer with identity between all pairs of nematodes examined. Proportion of match designated by shades of gray in scale at lower right. The tempo of sequence evolution differs between the proximal and distal portions of this molecule. This difference can be observed at all phylogenetic distances, from most distant (A-C) to least distant (D) and intermediate distances (E-F), though more conservation is observed between closer-related species (see Kiontke et al., 2004 for phylogeny).

Few motifs of 9 bp and none of greater than 9 bp found in the proximal *C. elegans* promoter also occur in the distal promoter (G). The same is true for *C. briggsae* with a window of 10 bp (H). Few shared motifs of 10 bp are identified in a comparison between *C. briggsae* distal *unc-47* promoter and the distal promoter sequence of an unrelated gene, the paramyosin *unc-15* (I). A similar number of off-diagonal matches are identified when orthologous distal *unc-47* promoter sequences are compared (A-F). Therefore, sequence conservation outside of the proximal *unc-47* promoters appears to be no greater than would be expected by chance. Dotplots were generated with the Mirror Tool of GeneGrokker v28.2D web server (https://chargaff.dartmouth.edu:1813).
