# Supplementary material for: Distinct Functional Constraints Partition Sequence Conservation in a cis-Regulatory Element
Source: PLoS Genet. 2011 Jun 2;7(6):e1002095. doi: 10.1371/journal.pgen.1002095 (PMC3107193; doi:10.1371/journal.pgen.1002095)
Supplement: Figure S3 — Integrated and extrachromosomal transgenes show corresponding effect of promoter length on pattern consistency. (PDF) [file pgen.1002095.s003.pdf]

Figure S3. Integrated and extrachromosomal transgenes show corresponding effect of promoter length on pattern consistency.

A

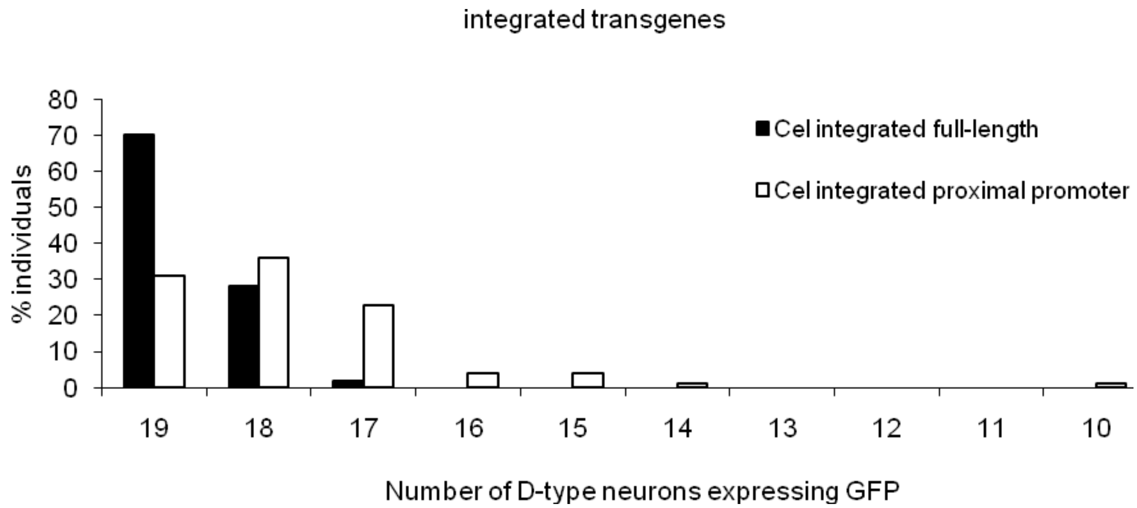

B

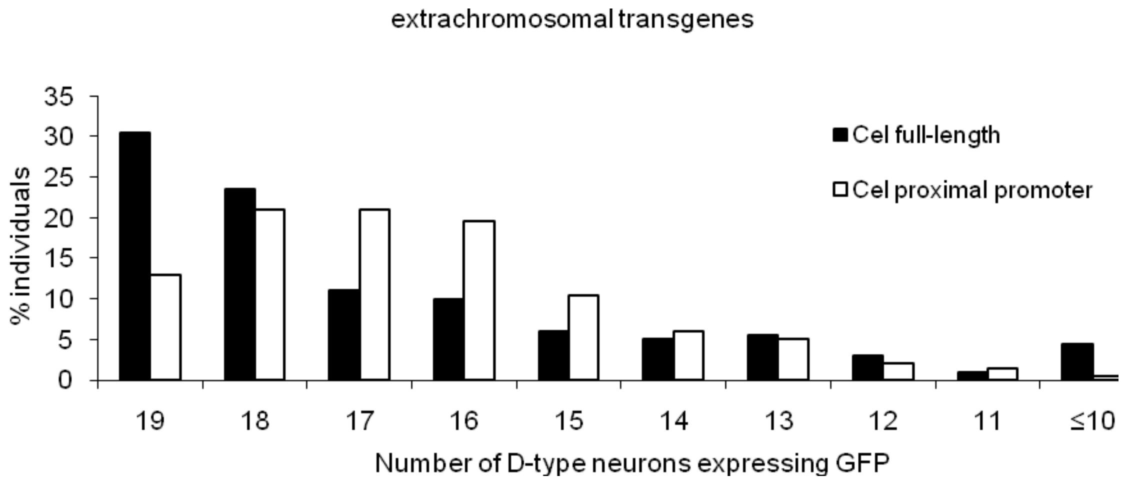

C. elegans full-length promoters drive more consistent expression than the proximal promoter whether introduced as integrated (A) or extrachromosomal transgenes (B). Wilcoxon tests for integrated full-length vs. proximal $p=1.9\times10^{-10}$; extrachromosomal full-length vs. proximal $p=3.2\times10^{-3}$. Integrated transgenes were more consistent overall. All counts were done in a blinded fashion. Because results were concordant between intergrated and extrachromosomal transgene experiments, we used extrachromosomal transgenes for the remainder of our study, as GFP expression was stronger overall and easier to measure.
