# Supplementary material for: Distinct Functional Constraints Partition Sequence Conservation in a cis-Regulatory Element
Source: PLoS Genet. 2011 Jun 2;7(6):e1002095. doi: 10.1371/journal.pgen.1002095 (PMC3107193; doi:10.1371/journal.pgen.1002095)
Supplement: Figure S4 — Multiple independent full-length and proximal strains are consistent in their robustness. (PDF) [file pgen.1002095.s004.pdf]

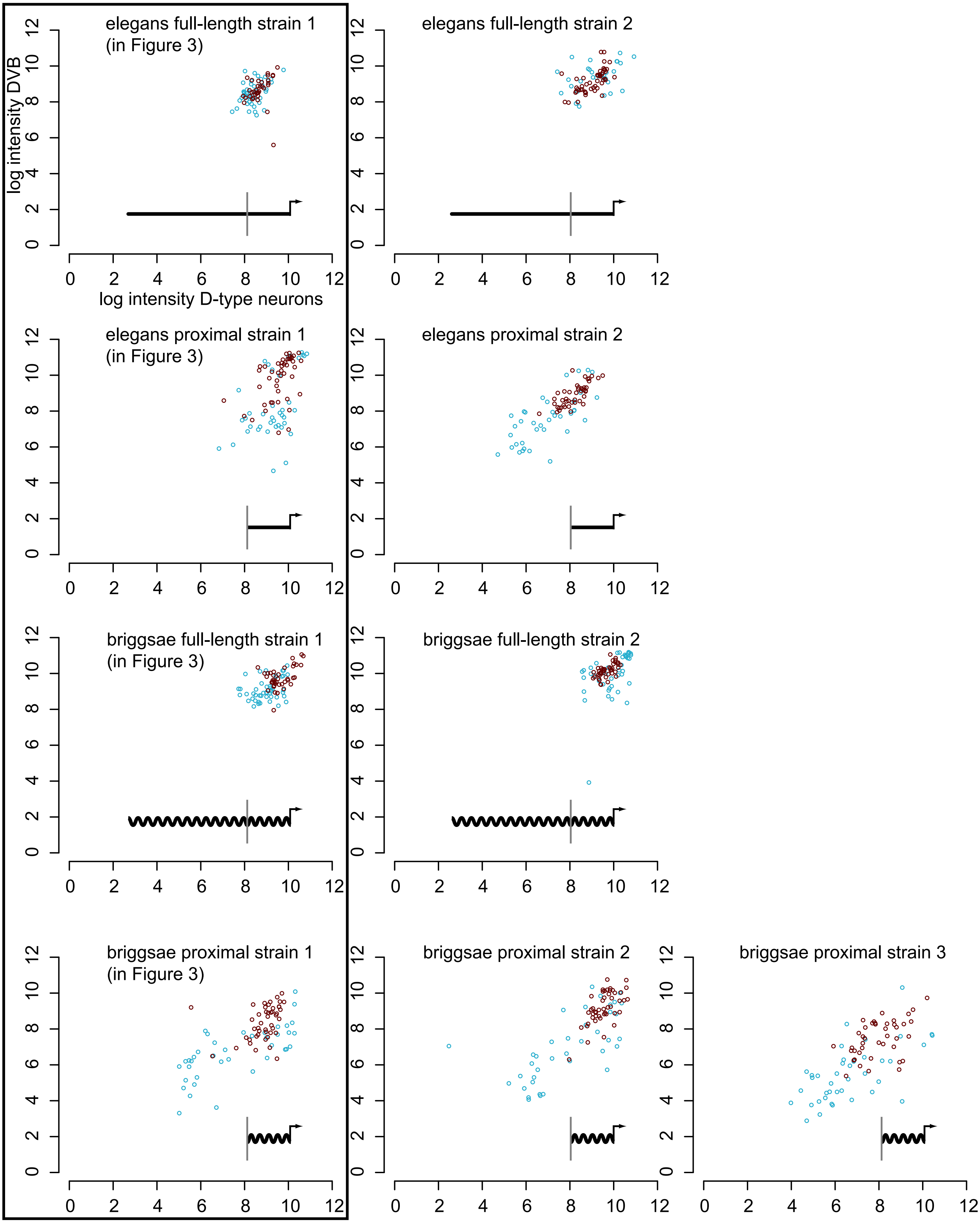

Figure S4. Multiple independent full-length and proximal strains are consistent in their robustness. Distribution of fluorescence intensity driven by *C. elegans* full-length and proximal, and *C. briggsae* full-length and proximal promoters in multiple independent strains. The boxed graphs are those presented in Figure 3.
