# Supplementary material for: Distinct Functional Constraints Partition Sequence Conservation in a cis-Regulatory Element
Source: PLoS Genet. 2011 Jun 2;7(6):e1002095. doi: 10.1371/journal.pgen.1002095 (PMC3107193; doi:10.1371/journal.pgen.1002095)
Supplement: Figure S5 — Multiple independent chimeric and deleted strains are consistent in their robustness. (PDF) [file pgen.1002095.s005.pdf]

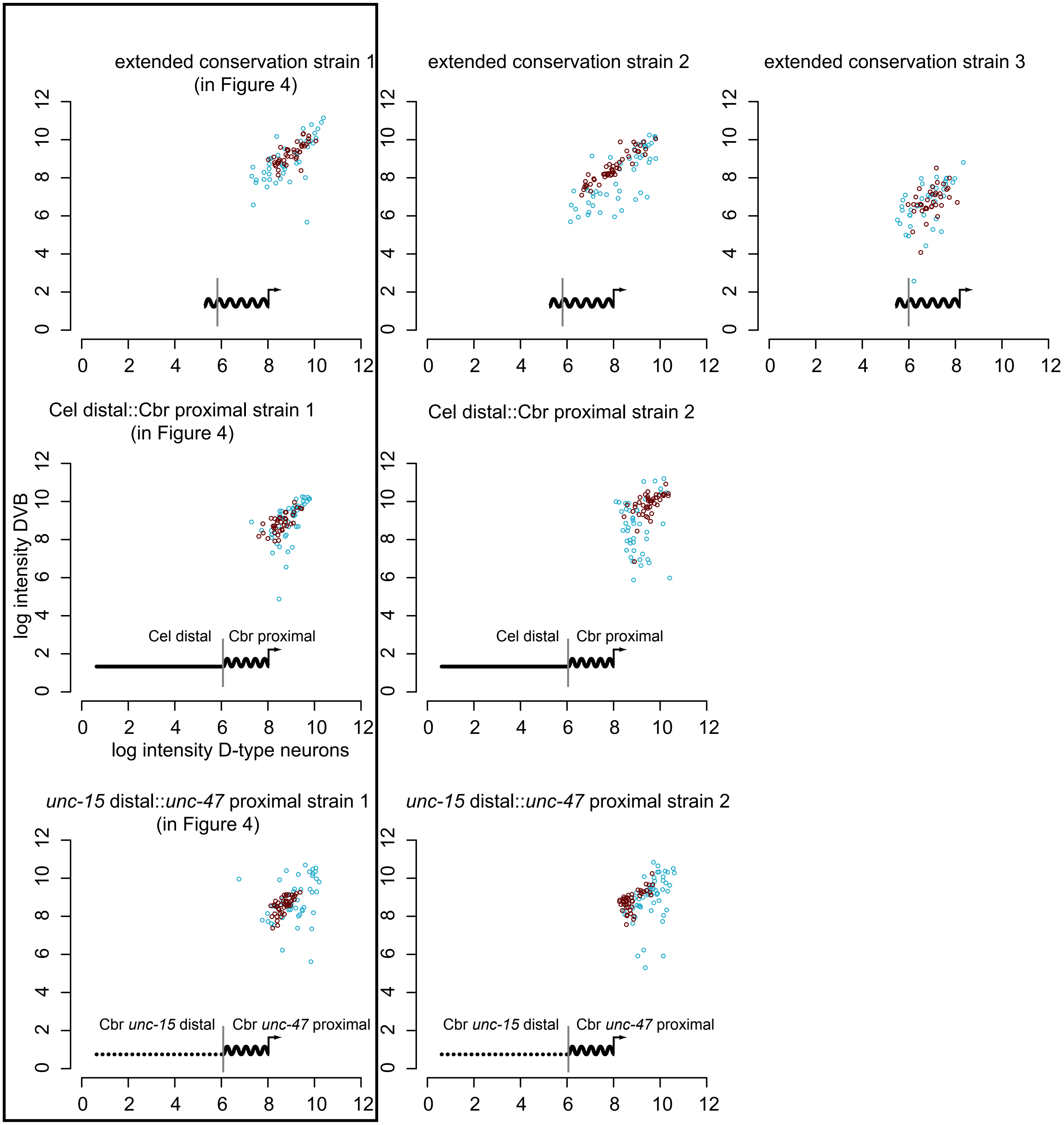

Figure S5. Multiple independent chimeric and deleted strains are consistent in their robustness.
Distribution of fluorescence intensity driven by *C. briggsae* extended conservation, chimeric *C. elegans* distal::*C. briggsae* proximal, and chimeric *C. briggsae unc-15* distal::*unc-47* proximal promoters in multiple independent strains. The boxed graphs are those presented in Figure 4.
