# Supplementary material for: Distinct Functional Constraints Partition Sequence Conservation in a cis-Regulatory Element
Source: PLoS Genet. 2011 Jun 2;7(6):e1002095. doi: 10.1371/journal.pgen.1002095 (PMC3107193; doi:10.1371/journal.pgen.1002095)
Supplement: Figure S6 — The C. briggsae unc-25 distal promoter confers robustness of expression pattern. (PDF) [file pgen.1002095.s006.pdf]

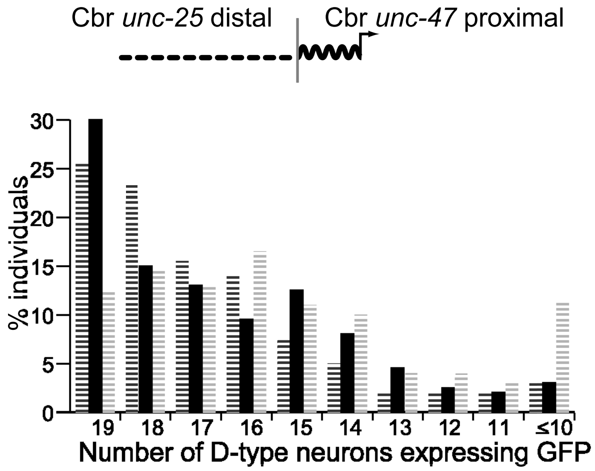

Figure S6. The ***C. briggsae unc-25*** **distal promoter confers robustness of expression pattern.** Percentage of 200 individuals expressing GFP in indicated number of D-type neurons from a chimeric promoter composed of distal *C. briggsae unc-25* sequence and the *C. briggsae unc-47* proximal promoter (black bars). For comparison, *C. briggsae unc-47* full-length and proximal promoters are shown in black and gray hashed bars, respectively.
