# Supplementary material for: Distinct Functional Constraints Partition Sequence Conservation in a cis-Regulatory Element
Source: PLoS Genet. 2011 Jun 2;7(6):e1002095. doi: 10.1371/journal.pgen.1002095 (PMC3107193; doi:10.1371/journal.pgen.1002095)
Supplement: Figure S7 — Nucleosome occupancy in robustness-conferring sequences. (PDF) [file pgen.1002095.s007.pdf]

A

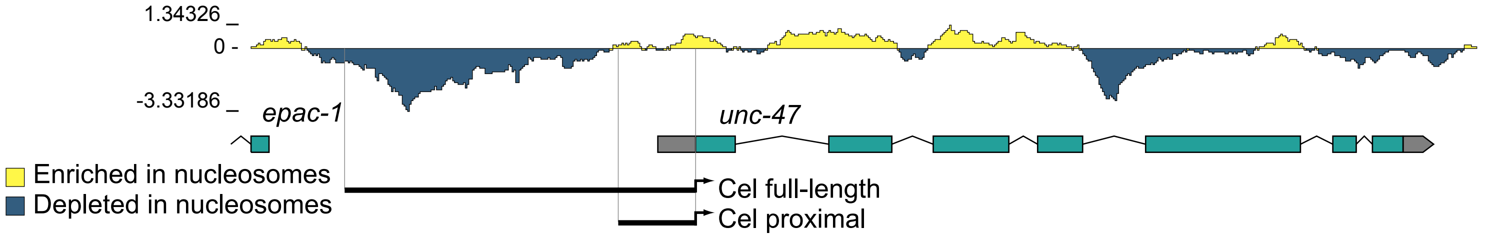

B

Over-represented in nucelosome cores	ACC	ACG	AGC	ATC	CAC	CAG	CCA	CCC	CCG	CCT	CGC	CGG	CGT	CTC	CTG	GAC	GCG	GGC	GCT	GTC	GTG	TCC	TCG	TCT	TGC	TGG			
Under-represented in nucleosome cores	AAA	AAT	ATA	ATT	GAA	TAA	TAT	TTA	TTT																				
Neither under- nor over-represented in nucleosome cores	AAC	AAG	ACA	ACT	AGA	AGG	AGT	ATG	CAA	CAT	CGA	CTA	CTT	GCG	GAT	GCA	GCG	GGA	GGG	GGT	GTA	GTT	TAC	TAG	TCA	TGA	TGT	TTC	TTG

C

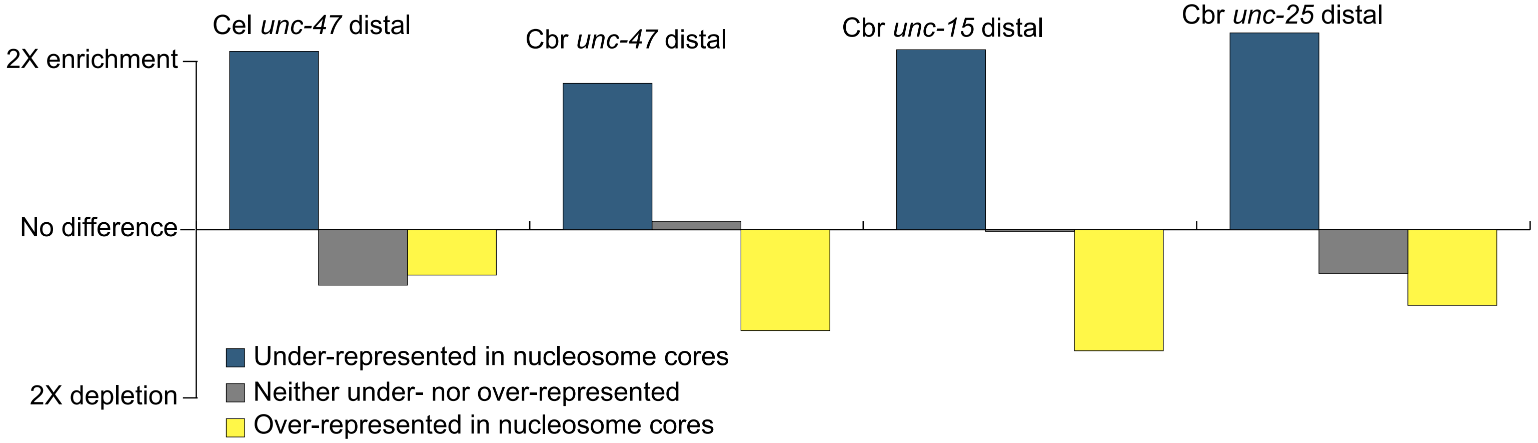

D

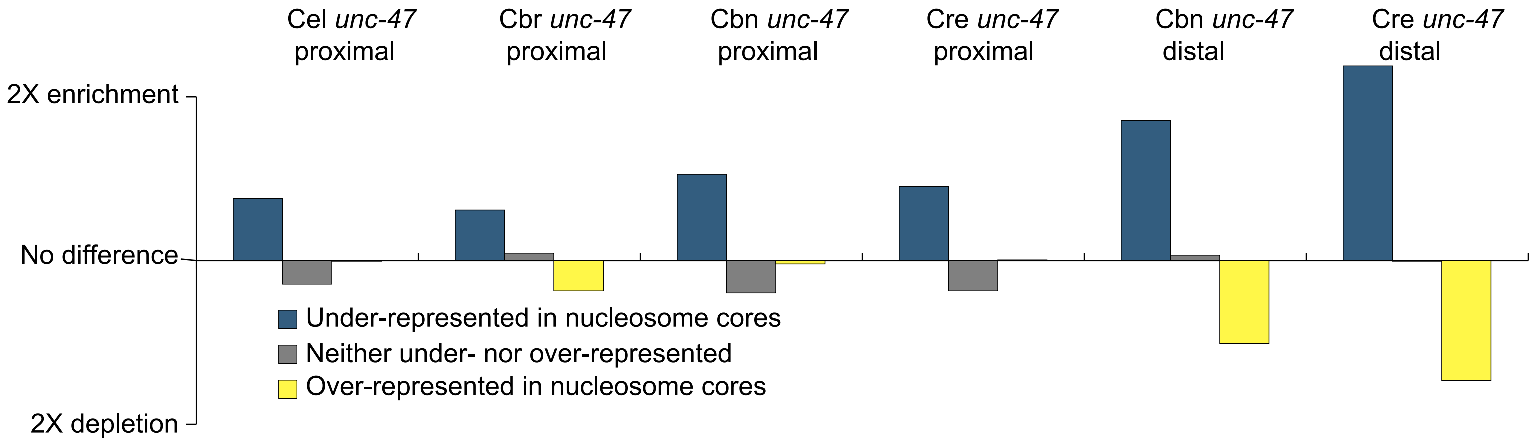

E

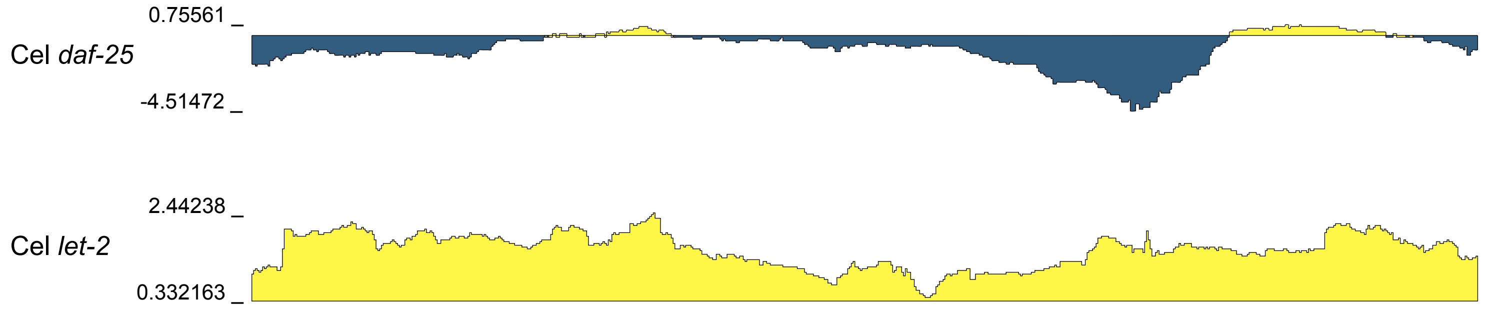

Figure S7. Nucleosome occupancy in robustness-conferring sequences.
(A) The *unc-47* locus of *C. elegans*, shown from the next annotated upstream gene (*epac-1*) to the end of *unc-47* transcript (teal boxes depict exons, lines depict introns, gray boxes depict UTRs). The extent of *C. elegans* full-length and proximal promoters used in experiments are shown with black bars. Blue and yellow colors represent nucleosome depletion and enrichment, respectively. The data are derived from the “adjusted nucleosome coverage” track as experimentally determined by Valouev et al. (2008) and accessed via UCSC genome browser. The scale of adjusted nucleosome coverage is indicated on the left. (B) Trinucleotide bins, based on the data presented in Valouev et al. (2008) Figure 7. (C) Enrichment/depletion of three categories of trinucleotides in four sequences that confer robustness relative to the sequence of the pPD95.75 vector (log scale). (D) Enrichment/depletion of three categories of trinucleotides in proximal and distal *unc-47* promoters from *C. elegans*, *C. briggsae*, *C. brenneri* and *C. remanei* relative to the sequence of the pPD95.75 vector (log scale). (E) Nucleosome occupancy of the 1kb fragment from the *C. elegans daf-25* locus used in the experiment shown in Figure 5B and of the 1kb fragment from the *C. elegans let-2* locus used in the experiment shown in Figure 5C.
