# Supplementary material for: Distinct Functional Constraints Partition Sequence Conservation in a cis-Regulatory Element
Source: PLoS Genet. 2011 Jun 2;7(6):e1002095. doi: 10.1371/journal.pgen.1002095 (PMC3107193; doi:10.1371/journal.pgen.1002095)
Supplement: Table S1 — Conservation of proximal promoters and distal sequence among four nematode species. (PDF) [file pgen.1002095.s008.pdf]

Table S1. Conservation of proximal promoters and distal sequence among four nematode species

Species	length (bp) of proximal promoter	base pairs of proximal sequence in blocks ≥10 nt 100% conserved with *C. elegans*	% proximal sequence in blocks ≥10 nt 100% conserved with *C. elegans*	length of distal sequence used in assays	base pairs of distal sequence in blocks ≥10 nt 100% conserved with *C. elegans*	% distal sequence in blocks ≥10 nt 100% conserved with *C. elegans*
C. briggsae	254	82	32.3%	1121	11	1.0%
C. brenneri	242	92	38.0%	730	0	0.0%
C. remanei	230	88	38.3%	923	11	1.2%
