# Supplementary material for: Distinct Functional Constraints Partition Sequence Conservation in a cis-Regulatory Element
Source: PLoS Genet. 2011 Jun 2;7(6):e1002095. doi: 10.1371/journal.pgen.1002095 (PMC3107193; doi:10.1371/journal.pgen.1002095)
Supplement: Table S2 — Comparisons of variation of all strains at 15°C and 26°C. (PDF) [file pgen.1002095.s009.pdf]

	Strain	Mean at 15°C	Variance at 15°C	Mean at 26°C	Variance at 26°C	K-S test p-value
C. elegans full-length	strain 1 (in Figure 3)	0.706	0.146	0.635	0.311	0.2403
	strain 2	1.020	0.340	0.771	0.154	0.0086
C. elegans proximal	strain 1 (in Figure 3)	1.756	1.023	1.215	0.478	0.0094
	strain 2	1.608	0.726	0.769	0.155	1.2×10^{-6}
C. briggsae full-length	strain 1 (in Figure 3)	0.762	0.151	0.680	0.163	0.0738
	strain 2	1.034*	0.170*	0.455	0.051	2.3×10^{-9}
C. briggsae proximal	strain 1 (in Figure 3)	2.188	0.848	1.025	0.415	2.8×10^{-9}
	strain 2	2.364	1.151	0.900	0.321	1.3×10^{-10}
	strain 3	1.947	1.325	1.279	0.364	0.0120
Cel distal::Cbr proximal	strain 1 (in Figure 4)	1.013	0.449	0.565	0.113	0.0020
	strain 2	1.302	0.556	0.619	0.244	2.3×10^{-5}
extended conservation	strain 1 (in Figure 4)	1.262	0.515	0.664	0.111	5.6×10^{-5}
	strain 2	1.689	0.385	0.933	0.384	2.7×10^{-4}
	strain 3	1.239	0.509	0.772	0.270	6.8×10^{-4}
unc-15 distal::*unc-47* proximal	strain 1 (in Figure 5)	1.164	0.475	0.495	0.078	5.2×10^{-7}
	strain 2	1.059	0.596	0.562	0.115	7.8×10^{-4}

*1.155351 and 0.7812112 if including outlier

Table S2. Comparisons of variation of all strains at 15°C and 26°C. Values in columns 3 to 6 represent the distribution of geometric distances between all data points for a particular strain/treatment and the mean of that strain/treatment. K-S=Kolmogorov – Smirnov test.
